# Fluoroscopy-Guided Lumbar Spinal Nerve Stimulation to Treat Chronic Scrotal Pain

**DOI:** 10.7759/cureus.42298

**Published:** 2023-07-22

**Authors:** Naeem Haider, Akshat Gargya

**Affiliations:** 1 Anesthesiology and Pain Medicine, University of Vermont Medical Center, Burlington, USA

**Keywords:** peripheral nerve stimulation, neuromodulation, chronic pain management, chronic scrotal pain, lumbar spinal nerve, fluoroscopy guided

## Abstract

Chronic scrotal pain (CSP) is a challenging problem for both pain physicians and urologists. Depending on the cause, treatment options may include pharmacological management, spermatic cord blocks, microsurgical denervation of the spermatic cord, botulinum toxin injections, and ultrasound-guided peripheral nerve stimulation (PNS) of ilioinguinal and iliohypogastric nerves. We describe a new target for the treatment of CSP by PNS of the L2 spinal nerve and a novel technical approach of using fluoroscopic guidance to stimulate lumbar spinal nerves, which can potentially be used for different indications.

## Introduction

Chronic scrotal pain (CSP) is a challenging problem for both pain physicians and urologists and accounts for 2-5% of all new outpatient urology visits [1]. It is poorly understood, and despite attempts at medical management, there are many patients for whom no satisfactory treatment is available [1-3]. It can significantly affect the quality of life of patients, and depressive symptoms are reported in over 40% of patients with CSP [4]. Treatment options depending on the cause may include pharmacological management, spermatic cord blocks, microsurgical denervation of the spermatic cord, Botox, peripheral nerve stimulation (PNS) of ilioinguinal and iliohypogastric nerves, and radical orchiectomy, amongst others [2,3,5]. The approach for ilioinguinal and iliohypogastric PNS for chronic groin and testicular pain has been described using ultrasound-guided techniques [6,7]. We describe a new target for the treatment of CSP by PNS of the L2 spinal nerve and a novel technical approach of using fluoroscopic guidance to stimulate lumbar spinal nerves, which can potentially be used for different indications.

## Case presentation

A 33-year-old male with no significant past medical history presented to our pain clinic for the management of right-sided scrotal pain. The pain started three years prior with no inciting event, was 4/10 on Numerical Rating Scale (NRS), and increased to 10/10 with minimal activity. It was sharp, and intermittently radiated to the anterior part of the right thigh. The patient noted worsening discomfort after holding his urine, heavy lifting, and ejaculating. He had no alleviating factors and denied any lower urinary tract symptoms or constipation but did report some difficulty obtaining an erection as well as a decline in libido, despite a normal testosterone level. He started taking tadalafil 5 mg daily one year before presentation, which helped in decreasing scrotal pain. An ultrasound done two years prior showed no abnormalities other than a small left varicocele. The physical exam was unremarkable. The patient was also using ibuprofen/acetaminophen as needed, methocarbamol 750 mg tablet three times daily, and naproxen 500 mg two times daily as needed with minimal improvement in pain. He previously had a spermatic cord block, performed by urology, which did not provide any pain relief. The patient was then scheduled for L2 peripheral nerve stimulator with SPRINT (SPR Therapeutics, Cleveland, Ohio, USA). He was seen two months after the procedure for lead removal and reported 1/10 pain on the NRS scale and reported significant functional improvement in his activities of daily living. The patient was subsequently followed up at five months and did not report testicular pain at that visit.

Technique

**Figure 1:**
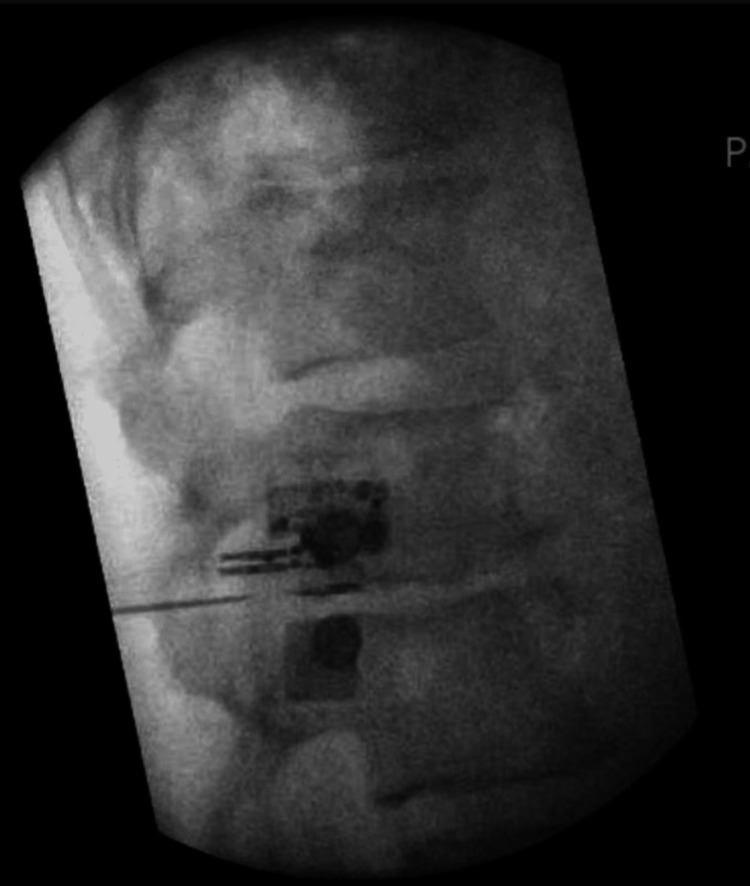
Lateral fluoroscopic image of the L2 vertebrae with stimulating needle outside and posterior to the neural foramen

**Figure 2:**
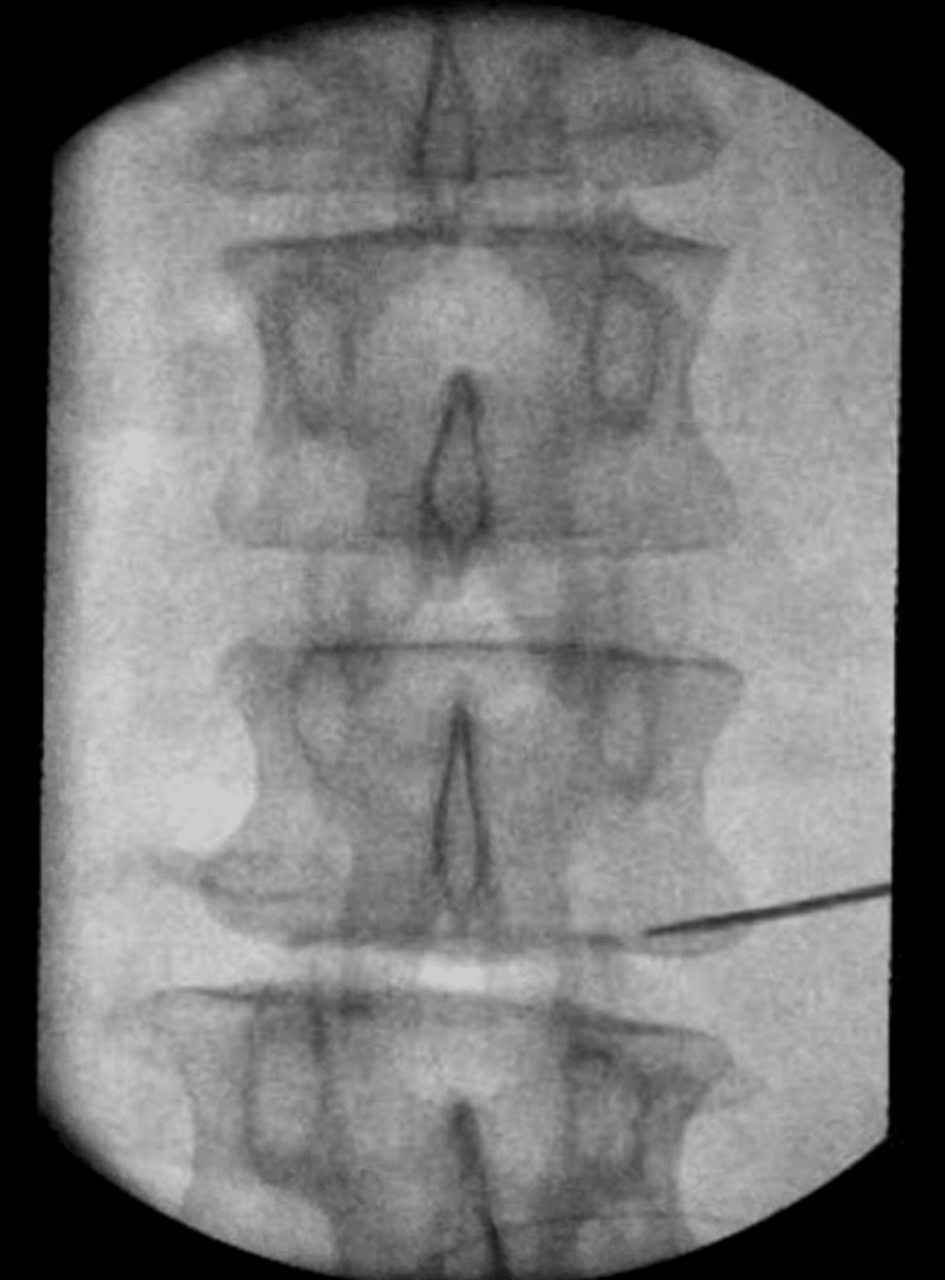
Anteroposterior fluoroscopic image of the L2 vertebrae showing the position of stimulating needle

The patient consented to the procedure and was positioned in a prone position on the table. Fluoroscopy was used to identify the L2 vertebral body. The skin overlying the area was cleaned with chlorhexidine. A right oblique view was obtained to visualize the right L2 transverse process. Skin and subcutaneous tissue were then injected with local anesthesia using 1% lidocaine. A 22 g Quincke 3.5-inch needle was used to anesthetize the track using 1% lidocaine. A 17G stimulating needle with stylet was placed along the anesthetized tract. A lateral view was obtained to determine the depth of the needle. The needle was advanced until it reached the outer intervertebral foramen and was stopped outside and posterior to the neuroforamen to be within 5-15 mm of the target L2 spinal nerve (Figure 1). An anteroposterior (AP) view was then obtained to verify the needle position (Figure 2).

**Figure 3:**
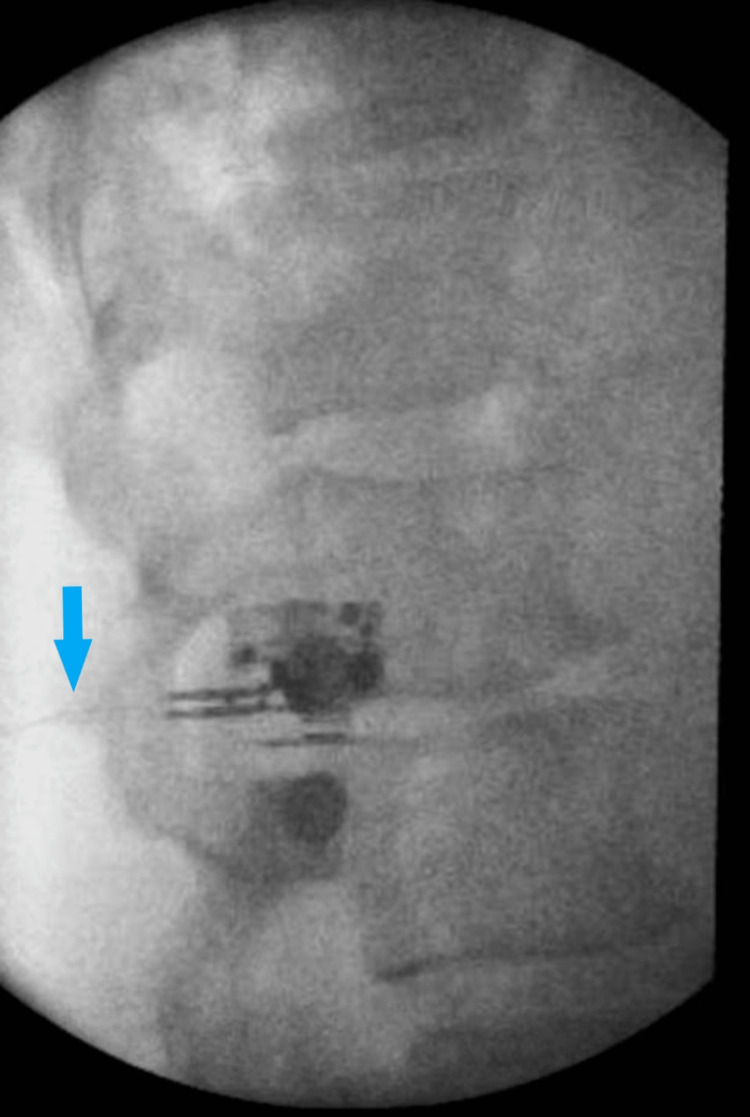
Lateral fluoroscopic image of L2 vertebrae with final lead placement (blue arrow)

**Figure 4:**
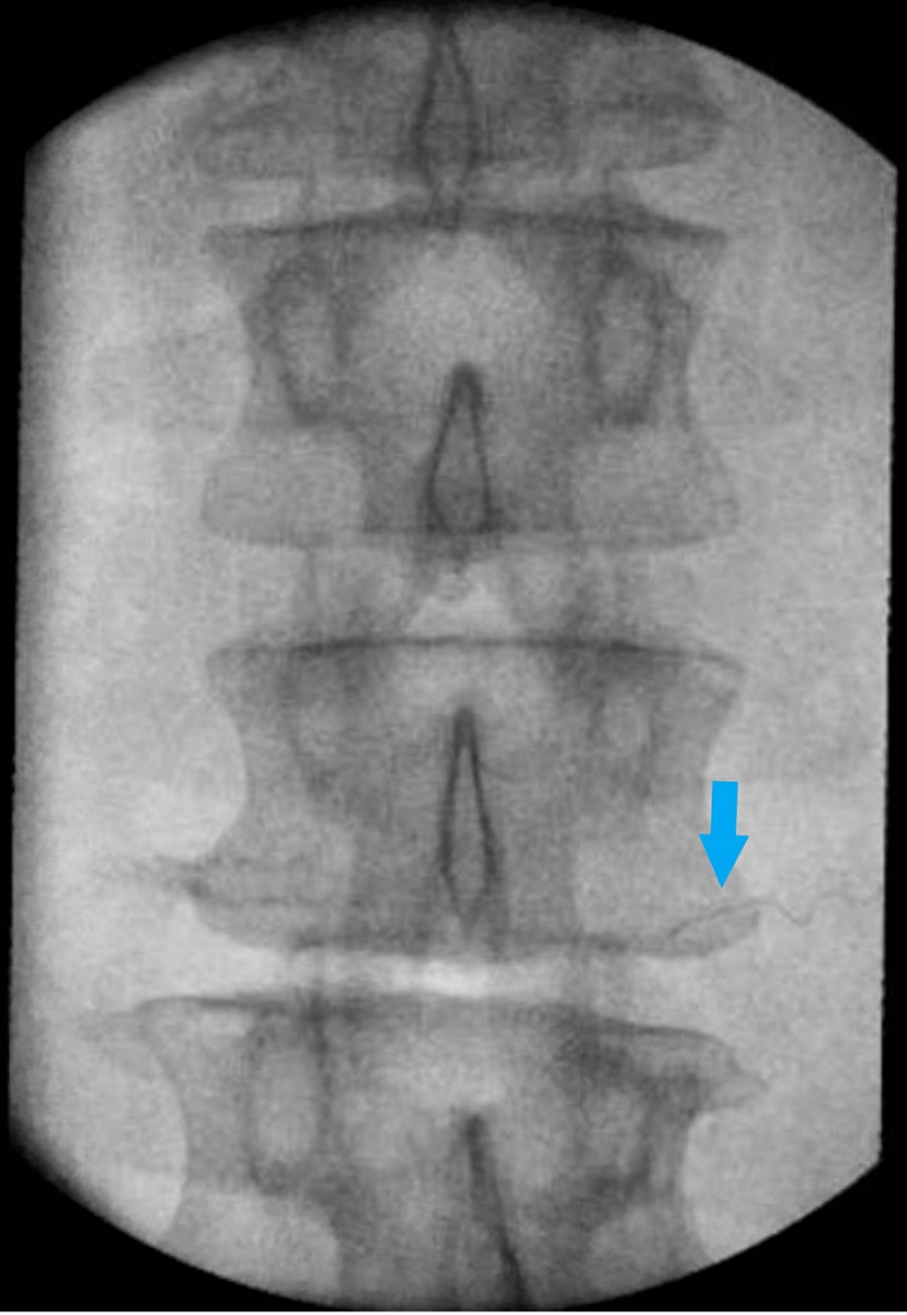
Anteroposterior fluoroscopic image of L2 vertebrae with final lead placement (blue arrow)

Successful stimulation was obtained by enabling selective activation of large-diameter sensory fibers, and the patient felt tingling in the groin in the region of pain at 60 milliamps. The stimulating stylet was removed, and a fine wire coiled lead was inserted. The lead was fixed in place with pressure as the introducer was removed. The final position of the lead was confirmed with successful stimulation by once again reproducing tingling in the region of pain and final AP and lateral fluoroscopic images were saved (Figures 3, 4).

Skin glue was used to fix the lead to the skin and a sterile occlusive dressing was applied. The lead was then attached to an external, wearable pulse generator mounted on the lateral side of the body using an adhesive hydrogel pad. The patient tolerated the procedure well and no apparent complications were reported by the patient.

## Discussion

Despite a thorough workup, 40% of patients with CSP have no identifiable etiology [8]. Management options are limited, and successful reduction of pain is hard to achieve. Spinal L2 PNS offers a novel and minimally invasive approach to managing CSP. In our patient, we achieved a significant reduction in pain comparable to published case reports of using ultrasound-guided PNS of the ilioinguinal nerves to manage groin and testicular pain [3]. Our case report introduces a new target for pain management in CSP. Additionally, we have proposed a novel technique that involves using fluoroscopy to stimulate lumbar spinal nerves.

PNS currently has various applications in adults, including complex regional pain syndrome (CRPS), peripheral neuropathy from post-herpetic neuralgia, chronic knee osteoarthritis, quadriceps tendon rupture, or neuropathic pain originating from ulnar, sciatic, or femoral nerves [9-14]. There are various mechanisms through which PNS may play a role in the reduction of pain. It acts both on the peripheral and central nervous systems, including critical cortical areas, and has been shown to modulate inflammatory and autonomic nervous system pathways [15-17]. PNS leads are left near the dorsal root ganglion and potentially cause ortho- and antidromic effects at the ganglion and the lumbar sympathetic chain, which are known to influence neuropathic pain [18]. In addition, since central sensitization causes the worsening of chronic pain and leads to a heightened response to pain, we believe by reduction of central sensitization, PNS of the L2 spinal nerve achieved a reduction in pain in our patient [4].

Often patients with CSP are prescribed oral medications like non-steroidal anti-inflammatory drugs (NSAIDs), antidepressants, and anticonvulsants, and neuropathic pain medications such as gabapentin and pregabalin, and suffer from side effects, including gastric ulcers, kidney disease, and mental cloudiness [1]. PNS is a non-pharmacological approach to treating CSP and can be implanted in an outpatient setting and avoids the side effects of pharmacological medications. It can also be used in combination with other treatments such as psychological support and medication management, and has the potential to avoid surgery. In our case, if the patient experiences suboptimal long-term benefit after lead removal, a permanent PNS can be considered after the 12-month washout period of pain relief from the SPRINT PNS. Further research to compare different approaches for the management of CSP is recommended.

The procedure for lumbar spinal nerve stimulation using ultrasound guidance has been described by Gargya et al. [19]. Ultrasound-guided stimulation requires a steep learning curve and needle visualization may be limited in technically challenging cases, especially in patients with large body habitus, patients with anatomical variation, and implanted hardware. For pain physicians, fluoroscopy is an easily available imaging modality and requires minimal learning. There are some limitations to fluoroscopy. Fluoroscopic guidance carries a risk of exposure to ionizing radiation and soft tissue, and vascular structure visualization can be challenging.

## Conclusions

In conclusion, fluoroscopy-guided L2 nerve stimulation is a novel technique and provides an alternative approach to pain management in patients with CSP. The technique of using fluoroscopy-guided lumbar spinal nerve stimulation can also potentially be used in the future for various other indications.
